# HIV Tat Protein Selectively Impairs CB_1_ Receptor-Mediated Presynaptic Inhibition at Excitatory But Not Inhibitory Synapses

**DOI:** 10.1523/ENEURO.0119-20.2020

**Published:** 2020-06-17

**Authors:** Mariah M. Wu, Stanley A. Thayer

**Affiliations:** 1 Graduate Program in Neuroscience, University of Minnesota Medical School, Minneapolis, MN 55455; 2 Department of Pharmacology, University of Minnesota Medical School, Minneapolis, MN 55455

**Keywords:** CB_1_ receptor, DSE, endocannabinoid, EPSC, HIV Tat, THC

## Abstract

Despite the success of antiretroviral therapy in suppressing viral load, nearly half of the 37 million people infected with HIV experience cognitive and motor impairments, collectively classified as HIV-associated neurocognitive disorders (HAND). In the CNS, HIV-infected microglia release neurotoxic agents that act indirectly to elicit excitotoxic synaptic injury. HIV trans-activator of transcription (Tat) protein is one such neurotoxin that is thought to play a major role in the neuropathogenesis of HAND. The endocannabinoid (eCB) system provides on-demand neuroprotection against excitotoxicity, and exogenous cannabinoids attenuate neurotoxicity in animal models of HAND. Whether this neuroprotective system is altered in the presence of HIV is unknown. Here, we examined the effects of Tat on the eCB system in rat primary hippocampal cultures. Using whole-cell patch-clamp electrophysiology, we measured changes in retrograde eCB signaling following exposure to Tat. Treatment with Tat significantly reduced the magnitude of depolarization-induced suppression of excitation (DSE) in a graded manner over the course of 48 h. Interestingly, Tat did not alter this form of short-term synaptic plasticity at inhibitory terminals. The Tat-induced decrease in eCB signaling resulted from impaired CB_1_ receptor (CB_1_R)-mediated presynaptic inhibition of glutamate release. This novel loss-of-function was particularly dramatic for low-efficacy agonists such as the eCB 2-arachidonoylglycerol (2-AG) and Δ^9^-tetrahydrocannabinol (Δ^9^-THC), the main psychoactive ingredient in marijuana. Our observation that HIV Tat decreases CB_1_R function *in vitro* suggests that eCB-mediated neuroprotection may be reduced *in vivo*; this effect of Tat may contribute to synaptodendritic injury in HAND.

## Significance Statement

Activation of the endocannabinoid (eCB) system protects against excitotoxicity. Whether this neuroprotection is altered in the presence of HIV is unknown. We show for the first time in an *in vitro* model of HIV neurotoxicity that an HIV protein selectively impairs eCB-mediated synaptic plasticity at excitatory but not inhibitory terminals. This selective effect of an HIV protein may unbalance synaptic networks, exacerbating the damage that underlies HIV-associated neurocognitive disorders (HAND). Thus, protecting or enhancing eCB signaling may attenuate the symptoms of HAND.

## Introduction

Nearly half of all HIV-positive individuals experience some degree of neurological impairment (Heaton et al., 1995; Tozzi et al., 2005; Bateup et al., 2013; Saylor et al., 2016). While antiretroviral therapy effectively suppresses viral replication, the prevalence of HIV-associated neurocognitive disorders (HAND) remains high and continues to be a significant public health burden. HAND symptoms range from subclinical cognitive impairment to debilitating dementia (Antinori et al., 2007), and their severity correlates with loss of synaptic markers (Ellis et al., 2007). Within the CNS, the hippocampus, prefrontal cortex, and striatum are particularly vulnerable to damage caused by HIV (Masliah et al., 1992; Wiley et al., 1998; Moore et al., 2006). Currently, there are no effective therapeutics to combat the neurological deficits seen in HAND patients.

HIV indirectly affects neurons, where infected microglia and macrophage release viral proteins, inflammatory cytokines, and excitotoxins resulting in synaptodendritic damage and altered network function (Ellis et al., 2007; Green et al., 2019). The HIV protein trans-activator of transcription (Tat) is a potent neurotoxin that evokes the loss of excitatory synapses (Kim et al., 2008; Shin and Thayer, 2013), an increase in the number of inhibitory synapses (Hargus and Thayer, 2013), and ultimately neuronal death (Eugenin et al., 2007). HIV Tat is present in the cerebrospinal fluid (Johnson et al., 2013), brain tissue (Hudson et al., 2000), and sera of HIV-infected individuals at concentrations ranging from 2 to 40 ng/ml (Xiao et al., 2000). Once viral DNA integrates into the host genome, Tat is continuously expressed, even in the presence of combined antiretroviral therapy (Johnson et al., 2013). The titer of antibodies against Tat negatively correlates with HAND symptoms (Bachani et al., 2013), suggesting that Tat accumulation is linked to cognitive deficits.

The endocannabinoid (eCB) system regulates many physiological processes of relevance to HAND including mood, anxiety, appetite, neuroinflammation, motor control, and neuroprotection (Rodríguez de Fonseca et al., 2005). The eCB system protects against excitotoxicity and attenuates epileptiform activity (Shen et al., 1996; Marsicano and Lutz, 1999; Nagayama et al., 1999). Notably, several studies using models of HAND have shown that pharmacological interventions targeted at the eCB system protect against HIV-mediated synaptodendritic damage. Cannabinoid type 2 receptor (CB_2_R) agonists attenuate neuroinflammation in a murine model of neuroAIDS (Gorantla et al., 2010) and protect human dopaminergic neurons from toxicity elicited by an HIV envelope protein (Hu et al., 2013). Application of the eCBs anandamide and 2-arachidonoylglycerol (2-AG) reduced HIV-induced increases in [Ca^2+^]_i_ through their actions on cannabinoid receptors *in vitro* (Xu et al., 2017). Similarly, pharmacological administration of exogenous cannabinoids or inhibitors of eCB metabolism attenuated neuronal damage elicited by HIV proteins (Kim et al., 2011a; Hermes et al., 2018; Zhang and Thayer, 2018). Thus, targeting the eCB system in HAND has therapeutic potential; it is less clear how the presence of HIV in the CNS affects eCB signaling.

Excitotoxic stimuli alter the eCB system. For example, febrile seizures elevate levels of the proinflammatory cytokine interleukin 1β (IL-1β), producing a long-lasting upregulation of eCB-mediated inhibition of GABAergic transmission, resulting in a subsequent increased susceptibility to seizure (Chen et al., 2003; Feng et al., 2016). Interestingly, eCB modulation of glutamatergic transmission was not affected in this model, setting a precedent for differential modulation of eCB signaling at inhibitory versus excitatory synapses under neuroinflammatory conditions. In transgenic mice expressing HIV Tat, CB_1_Rs are upregulated (Jacobs et al., 2019); whether this increase preferentially affects GABAergic neurons is unclear.

To address this question, we examined the effects of HIV Tat on eCB-mediated retrograde signaling. Treating rat primary hippocampal cultures with Tat reduced the magnitude of depolarization-induced suppression of excitation (DSE) over the course of 48 h. Tat impaired CB_1_R-mediated presynaptic inhibition of glutamate release, but not GABA release. These results suggest that exposure to the HIV protein Tat may reduce neuroprotection mediated by the eCB system and alter the sensitivity of excitatory synaptic networks to cannabinoids.

## Materials and Methods

### Materials

Materials were obtained from the following sources: 6-cyano-7-nitroquinoxaline-2,3-dione (CNQX), (2R)-amino-5-phosphonovaleric acid (APV), bicuculline methochloride, Win55212-2 (Win-2), and SR141716A were obtained from Tocris Biosciences; 2-AG and JZL184 were obtained from Cayman Chemical; DMEM, fetal bovine serum, and horse serum were obtained from Invitrogen; (S)−3,5-dihydroxyphenylglycine (DHPG) and all other chemicals were obtained from Sigma-Aldrich. A plasmid encoding the tandem C1 domains (C1ab) of PKD fused to GFP (pCMV-PKDC1ab-GFP) was kindly gifted by Tamas Balla (Kim et al., 2011b), National Institute of Child Health and Human Development. HIV-1 Tat_1-86_ (Clade B, recombinant) was obtained through the National Institutes of Health (NIH) AIDS Research and Reference Reagent Program, Division of AIDS, National Institute of Allergy and Infectious Diseases from John Brady. Δ^9^-tetrahydrocannabinol (Δ^9^-THC) was obtained from the National Institute on Drug Abuse Drug Supply Program (Research Triangle Institute).

### Cell culture

The hippocampus is particularly vulnerable to the neurotoxic effects of HIV (Ellis et al., 2007); thus, we chose rat hippocampal neurons grown in primary culture for this study. Cultures were prepared as previously described (Waataja et al., 2008). Briefly, maternal Sprague Dawley rats were euthanized by CO_2_ inhalation under a protocol approved by the University of Minnesota Institutional Animal Care and Use Committee in accordance with the *NIH Guide for the Care and Use of Laboratory Animals*. Male and female fetuses were removed on embryonic day 17, and hippocampi were dissected and placed in Ca^2+^-free and Mg^2+^-free HEPES-buffered HBSS. Cells were dissociated by manual trituration using flame-narrowed Pasteur pipettes of decreasing aperture and resuspended in DMEM without glutamine supplemented with 10% fetal bovine serum and penicillin/streptomycin (100 units/ml and 100 μg/ml, respectively). Dissociated cells were then plated at a density of 60,000–80,000 cells per dish on either a 35-mm Petri dish with a 10-mm cover glass bottom (MatTek) or a 25-mm-round cover glass (#1) precoated with Matrigel (150 μl, 0.2 mg/ml). Neurons were grown in a humidified atmosphere of 10% CO_2_ and 90% air held at 37°C in an incubator. Cells were fed on days 1 and 7 by exchanging 75% of the medium with DMEM supplemented with 10% horse serum and penicillin/streptomycin. Cultures used in this study contained a mixture of 18 ± 2% neurons, 70 ± 3% astrocytes, and 9 ± 3% microglia (Kim et al., 2011a). Cells were grown for 12–13 d *in vitro* (DIV).

### Electrophysiology

Synaptic currents were recorded using the whole-cell configuration of the patch-clamp technique. Pipettes were pulled using a horizontal micropipette puller (P-87, Sutter Instruments) from glass capillaries with an outer diameter of 1 mm (Sutter Instrument) and pipette resistances of 3–5 MΩ. Membrane potential was held at −70 mV, and monosynaptic EPSCs/IPSCs were evoked with a bipolar platinum electrode (FHC) placed near a presynaptic neuron. DSE was evoked by a 15-s depolarization to 0 mV, which was previously shown to produce 50% DSE (Roloff et al., 2010). Each cell was exposed to a single stimulus. For DSE recordings pipettes were filled with the following intracellular solution: 120 mm potassium gluconate, 15 mm KCl, 6 mm MgCl_2_, 0.2 mm EGTA, 10 mm HEPES, and 5 mm Na_2_ATP, pH 7.3 with KOH, 290 mOsm/kg. Metabotropic suppression of excitation (MSE) was evoked by bath application of the selective group 1 metabotropic glutamate receptor (mGluR) agonist DHPG (1 μm). For MSE recordings, pipettes were filled with the following: 113 mm potassium gluconate, 15 mm KCl, 6 mm MgCl_2_, 10 mm BAPTA tetrapotassium, 10 mm HEPES, 5 mm Na_2_ATP, and 6 mm CaCl_2_, pH 7.2 with KOH, 290 mOsm/kg. Depolarization-induced suppression of inhibition (DSI) was evoked by a 2-, 5-, or 15-s depolarization to 0 mV (Ohno-Shosaku et al., 2001; Wilson and Nicoll, 2001; Isokawa and Alger, 2005). Most cells were exposed to a single stimulus. Some cells from both control and Tat-treated groups were exposed to up to three different stimulus strengths (2, 5, 15 s); there was a 6-min break between administration of serial depolarizing stimuli. For DSI recordings pipettes were filled with the following: 140 mm KCl, 0.2 mm EGTA, 10 mm HEPES, 10 mm glucose, 5 mm MgATP, and 0.3 mm Na_2_GTP, pH 7.2 with KOH, 290 mOsm/kg. All recordings were performed at room temperature in an extracellular solution composed of the following: 140 mm NaCl, 5 mm KCl, 9 mm CaCl_2_, 6 mm MgCl_2_, 5 mm glucose, and 10 mm HEPES, pH 7.4 with NaOH, 325 mOsm/kg. For EPSC recordings, 10 μm bicuculline methochloride was added to the extracellular recording solution; for IPSC recordings, 10 μm CNQX and 50 μm APV were added. Solutions were applied by a gravity-fed superfusion system. Whole-cell currents were amplified with an AxoPatch 200B (Molecular Devices), low-pass filtered at 2 kHz, and digitized at 10 kHz with a Digidata 1322A (Molecular Devices) digitizer and pClamp v.9.2 software (Molecular Devices). Voltage pulses (0.1 ms) were applied at a fixed rate of 0.5 Hz for all experiments unless otherwise specified using a Grass S44 stimulator with a SIU-5 stimulus isolation unit (Grass Instruments).

### Transfection

Rat hippocampal neurons were transfected on DIV10 or DIV11 with plasmid DNA using the calcium phosphate procedure described by Hargus and Thayer (2013). Briefly, hippocampal neurons were incubated for 30 min in DMEM supplemented with 1 mm kynurenic acid, 10 mm MgCl_2_, and 5 mm HEPES to reduce neurotoxicity. A DNA/calcium phosphate precipitate containing 1 μg of total plasmid DNA per well was prepared, allowed to form for 60 min at room temperature, and added to the culture. After a 60-min incubation period, cells were washed once with DMEM supplemented with MgCl_2_ and HEPES, then returned to conditioned media that had been saved at the beginning of the procedures.

### Confocal microscopy and image processing

Cultures grown in MatTek glass-bottom Petri dishes were transferred to the motorized stage of a Nikon A1 laser-scanning confocal microscope 48 h after transfection with pCMV-PKDC1ab-GFP and viewed through a 60× oil-immersion objective. The objective was focused to the middle of the soma in the *z*-dimension and held constant using the autofocus feature of the microscope. PKDC1ab-GFP was excited at 488 nm, and emission was collected at 500–550 nm. After recording (1 Hz) baseline images for 60 s, DHPG (1 μm) or vehicle was added directly to the media bathing the cell.

### Experimental design and statistical analysis

All data are presented as mean ± SEM. Electrophysiology data were analyzed using pCLAMP v.9.2. Live-cell imaging data were analyzed using NIS Elements and ImageJ. To minimize the effect of variables except the independent variable, experimental controls were run in parallel in all experiments. Only experiments with proper vehicle and/or drug controls were included in statistical analyses. For all experiments, an individual sample (*n *=* *1) is defined as a single cell from a single coverslip/Petri dish. To account for inherent variability across primary cultures from week to week, each experiment was replicated over at least three separate cultures with a sample size of at least six cells/group. Because primary neuronal cultures are derived from a mixture of male and female rat embryos, the culture preparation protocol ensures an unbiased evaluation across both sexes. Hypothesis testing was performed with Prism 8 (GraphPad Software). Data were tested for normality using Bartlett’s test. Experiments with two groups and one factor were analyzed using an unpaired two-tailed Student’s *t* test. Samples with unequal variance were analyzed with Welch’s two-tailed *t* test. Experiments with 3+ groups and one factor were analyzed using a one-way ANOVA followed by Tukey’s *post hoc* test. Experiments with 2+ groups and two factors were analyzed using a two-way ANOVA. Statistical significance was defined as *p *<* *0.05. OriginLab 2019 was used for curve-fitting decay rate and concentration-response data. Unless derived from curve fitting, confidence intervals (CIs) were determined using bootstrap resampling by uploading the same raw data used for hypothesis testing to https://www.estimationstats.com. The results of statistical analyses are presented in the figure legends and Table 1.

**Table 1:** Statistics table

	Figure	Group comparison	Datastructure	Type of test	*p* value	Effectsize	95% CI (lower toupper bound)
*a*	1*F*	Treatment	Normal	One-way ANOVA	<0.0001		
		Control vs Tat (4 h)		Tukey’s *post hoc*	0.5864^*^	–11.8% DSE	–22.5 to 0.13
		Control vs Tat (24 h)		Tukey’s *post hoc*	0.0002^*^	–41.8% DSE	–55.6 to –27.4
		Control vs Tat (48 h)		Tukey’s *post hoc*	<0.0001^*^	–46.8% DSE	–60.7 to –32.8
		Control vs hi-Tat (24 h)		Tukey’s *post hoc*	0.9759^*^	4.6% DSE	–9.06 to 19.6
		hi-Tat (24 h) vs Tat (24 h)		Tukey’s *post hoc*	<0.0001^*^	–46.5% DSE	–63.2 to –30.2
		hi-Tat (24 h) vs Tat (48 h)		Tukey’s *post hoc*	<0.001^*^	–51.5% DSE	–68.7 to –36.6
*b*		EPSC: control vs Tat (24 h)	Normal	Unpaired *t* test (two-tailed)	0.3641	–35.6 pA	–124.1 to 21.3
		IPSC: control vs Tat (24 h)	Normal^#^	Welch’s *t* test (two-tailed)	0.3406	–97.5 pA	–329.3 to 68.3
*c*	2*D*	Depolarization duration × Tat treatment	Normal	Two-way ANOVA: interaction	0.9182		
		Depolarization duration		Factor	0.0007		
		Tat treatment		Factor	0.9365		
*d*	3*C*	Control vs Tat (24 h)	Normal	Unpaired *t* test (two-tailed)	0.0001	–24.5% MSE	–31.2 to –18.5
*e*	4*C*	Tau: control vs Tat (24 h)	Normal	Unpaired *t* test (two-tailed)	0.7611	–0.4361 s	–3.554 to 2.680§
		Peak: control vs Tat (24 h)	Normal	Unpaired *t* test (two-tailed)	0.8827	–0.0411	–0.646 to 0.564§
*f*	5*C*	2-AG EC_50_: control vs Tat (24 h)	Normal^#^	Welch’s *t* test (two-tailed)	<0.0001	1.22 µM	0.716 to 1.724§
	5*D*	JZL184 treatment × Tat treatment	Normal	Two-way ANOVA: interaction	0.8380		
		JZL184 treatment		Factor	0.3572		
		Tat treatment		Factor	<0.0001		
*g*	6*D*	Win-2 EC_50_: control vs Tat (24 h)	Normal	Unpaired *t* test (two-tailed)	0.93	–0.16 nM	–3.81 to 4.13§
*h*	7*D*	[THC] × Tat treatment	Normal	Two-way ANOVA: interaction	0.9165		
		Tat treatment		Factor	<0.0001		
		[THC]		Factor	0.0111		
*i*		RAP (24 h) × Tat (24 h)	Normal	Two-way ANOVA: interaction	0.6978		
		RAP (24 h) treatment		Factor	0.9528		
		Tat (24 h) treatment		Factor	<0.0001		
*j*	8*B*	CPA: control vs Tat (24 h)	Normal	Unpaired *t* test (two-tailed)	0.6862	2.65%	–9.05 to 13.9
	8*C*	CAP: control vs Tat (24 h)	Normal	Unpaired *t* test (two-tailed)	0.8557	–1.83%	–21.9 to 14.7

Effect size is calculated as the mean difference.

* *p* values have been corrected for multiple comparisons.

# unequal variance.

^§^CI derived from error associated with curve fit.

## Results

### HIV Tat inhibits DSE in a time-dependent manner

**Figure 1.**
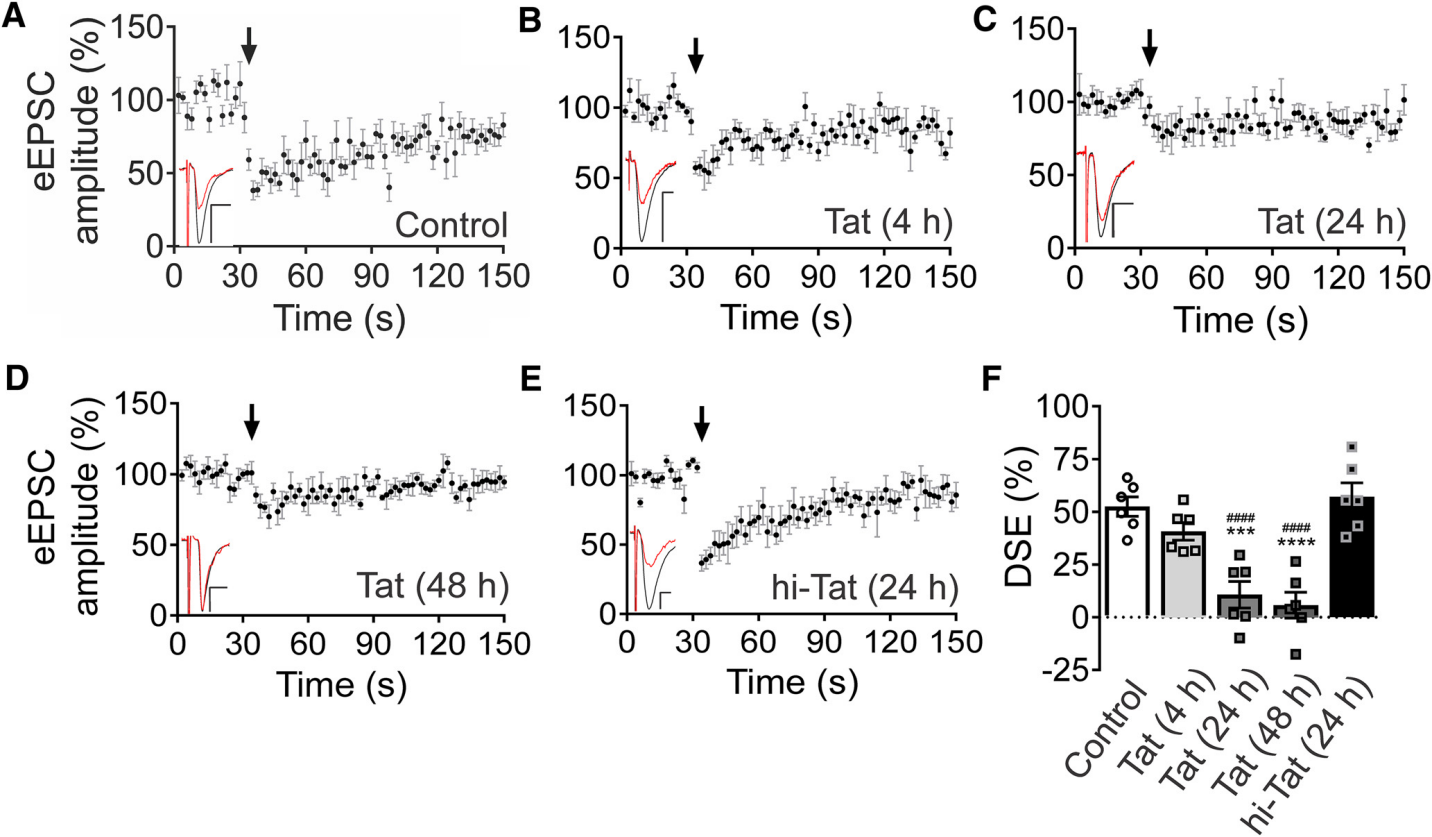
HIV Tat inhibits DSE in a time-dependent manner. ***A–E***, EPSCs were evoked at 0.5 Hz by stimulation of a presynaptic neuron with an extracellular electrode and recorded from a postsynaptic cell voltage-clamped at −70 mV. DSE was evoked by depolarizing the postsynaptic cell to 0 mV for 15 s at the time indicated by the arrow. Plots show mean EPSC amplitudes as a percentage of baseline (15 responses before depolarization) over time. Cultures were untreated (***A***, control) or treated with 50 ng/ml Tat for (***B***) 4, (***C***) 24, or (***D***) 48 h. ***E***, Treatment with heat-inactivated Tat (hi-Tat, 90°C for 30 min) for 24 h did not affect DSE. Insets show representative EPSC trace. Black: baseline, average of 15 responses before depolarization. Red: average of two responses after depolarization. Scale bars: 10 ms (horizontal bar) and 50 pA (vertical bar). ***F***, Summary bar graph shows mean magnitude of DSE for control (open bar) and Tat-treated groups (gray bars). Percentage DSE was calculated according to the equation: DSE (%) = 100*(ESPC_baseline_ – EPSC_DSE_)/EPSC_baseline_; EPSC_baseline_: average amplitude of 15 responses immediately before depolarization; EPSC_DSE_: average amplitude of two responses immediately after depolarization. One-way ANOVA (*F*_(4,25)_ = 17.9, *p *<* *0.0001; *n *=* *6 cells/group) was followed with Tukey’s *post hoc* test (***p <* *0.001, ****p <* *0.0001 relative to control; ^####^*p *<* *0.0001 relative to hi-Tat). Data are represented as mean ± SEM.

Depolarization of a postsynaptic hippocampal neuron evokes Ca^2+^ influx through voltage-gated Ca^2+^ channels that induces production of the eCB 2-AG (Ohno-Shosaku et al., 2001; Wilson and Nicoll, 2001). 2-AG then traverses the synaptic cleft in a retrograde manner to activate presynaptic CB_1_Rs, resulting in inhibition of glutamate transmission (Straiker and Mackie, 2005). We studied this process in primary rat hippocampal cultures following exposure to the HIV Tat_1-86_ (Clade B) protein. We used the whole-cell configuration of the patch-clamp technique to voltage-clamp a hippocampal neuron at −70 mV. ESPCs were evoked by a concentric bipolar stimulating electrode positioned near a presynaptic neuron. After recording a stable baseline, DSE was evoked by depolarizing the postsynaptic cell to 0 mV for 15 s (Fig. 1*A*; Roloff et al., 2010). Immediately following depolarization, the EPSC amplitude was inhibited by 53 ± 5% (Fig. 1*A*,*F*). DSE was completely blocked by pretreatment with the CB_1_R inverse agonist SR141716A (100 nm, *n *=* *4). To determine whether HIV Tat affects eCB signaling, we assessed DSE in Tat-treated cultures (50 ng/ml). Treatment with Tat for 4 h did not significantly affect DSE (Fig. 1*B*,*F*). DSE was significantly reduced following 24 (11 ± 6%; Fig. 1*C*,*F*) and 48-h Tat treatment (6 ± 6%; Fig. 1*D*,*F*; Table 1a). EPSC amplitude was not affected by 24-h Tat treatment (control = −147 ± 20 pA, Tat = −182 ± 31 pA; *t*_(24)_ = 0.925, *p *=* *0.36; Table 1b). To confirm that the effect of Tat required structurally intact protein, we treated cells with heat-inactivated Tat (hi-Tat; Fig. 1*E*) for 24 h and assessed DSE. Exposure to hi-Tat did not affect DSE, which was comparable to control (57 ± 7%; Fig. 1*E*,*F*). We conducted a subsequent analysis to determine effect size and precision. Tat treatment for 24 h was estimated to decrease DSE relative to control by 42%. Using bootstrap resampling, we calculated the 95% CI for this effect as 27–56% (Table 1a).

### Tat does not affect DSI

**Figure 2.**
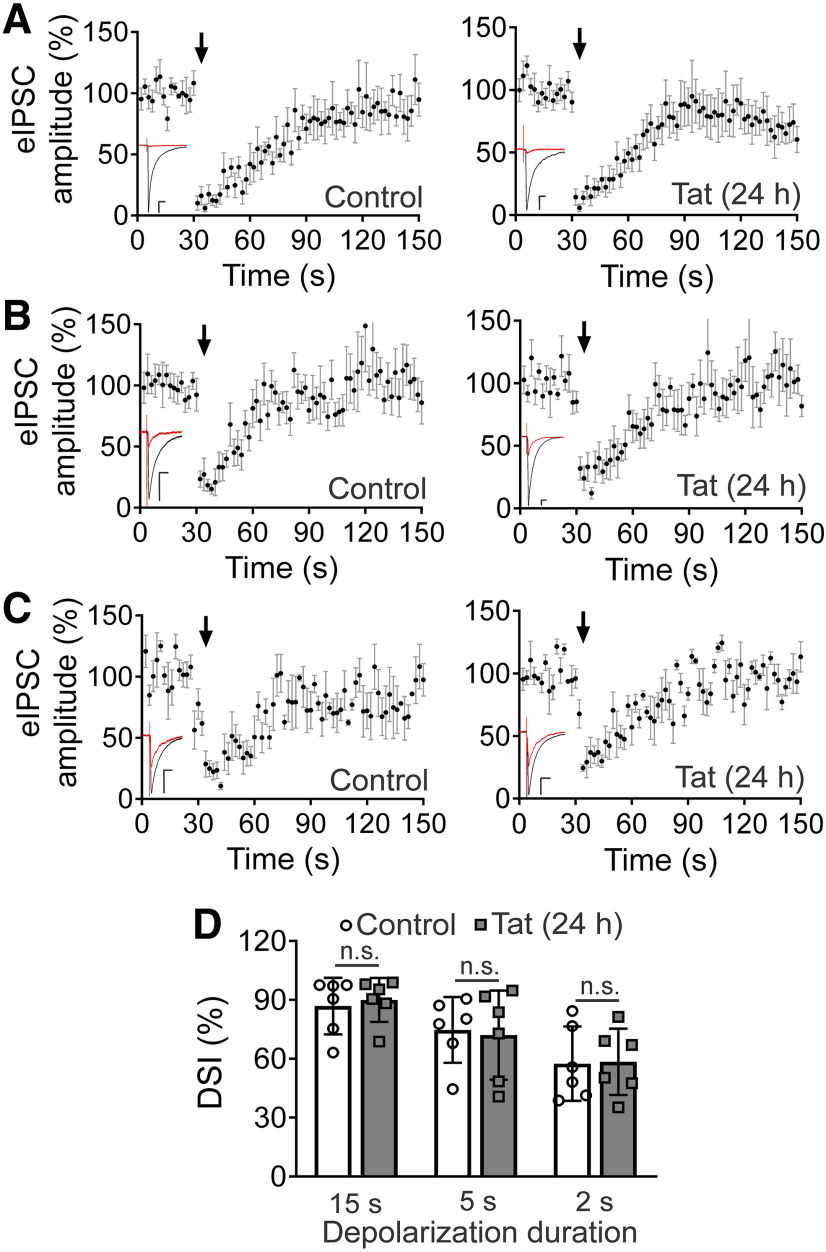
HIV Tat does not affect DSI. ***A–C***, IPSCs were recorded from a neuron held at −70 mV and evoked at 0.5 Hz by stimulation of a presynaptic neuron with an extracellular electrode. Cultures were either untreated (control) or treated with 50 ng/ml Tat for 24 h. The postsynaptic cell was depolarized to 0 mV for (***A***) 15, (***B***) 5, and (***C***) 2 s at the time indicated by the arrow. Plots show mean IPSC amplitudes as a percentage of baseline (15 responses before depolarization) over time. Insets show representative IPSC trace. Black: baseline, average of 15 responses before depolarization. Red: average of two responses after depolarization. Scale bars: 25 ms (horizontal bar) and 100 pA (vertical bar). ***D***, Summary bar graph shows the magnitude of DSI for control (open bars) and 24-h Tat-treated groups (gray bars). Percentage DSI was calculated according to the equation: DSI (%) = 100*(ISPC_baseline_ – IPSC_DSI_)/IPSC_baseline_; IPSC_baseline_: average amplitude of 15 responses immediately before depolarization; IPSC_DSI_: average amplitude of two responses immediately after depolarization. A two-way ANOVA found no interaction (depolarization duration × Tat treatment; *F*_(2,30)_ = 0.086, *p *=* *0.92, *n *=* *6 cells/group). Depolarization duration had a significant effect on DSI (*F*_(2,30)_ = 9.4, *p *=* *0.0007); n.s. = no significant difference. Data are represented as mean ± SEM.

Because CB_1_Rs are also present on inhibitory terminals (Katona et al., 1999), depolarization-induced 2-AG production also inhibits GABAergic transmission (Ohno-Shosaku et al., 2001; Wilson and Nicoll, 2001). To test whether Tat (50 ng/ml, 24 h) similarly affects DSI, we recorded evoked IPSCs, then depolarized the postsynaptic neuron to 0 mV. A 15-s depolarization produced 87 ± 6% inhibition of IPSC amplitude, consistent with the high density of CB_1_Rs on a subset of hippocampal interneurons (Katona et al., 1999). In cultures treated with Tat, DSI was unaffected (90 ± 5%; Fig. 2*A*,*D*). To confirm that we had not saturated DSI, we reduced the stimulus strength by shortening the duration of depolarization (Ohno-Shosaku et al., 2001; Wilson and Nicoll, 2001; Isokawa and Alger, 2005). Untreated cultures depolarized to 0 mV for 5 s exhibited 75 ± 7% inhibition; Tat treatment did not alter DSI evoked by a 5-s depolarization (72 ± 9%; Fig. 2*B*,*D*). A 2-s depolarization in untreated cells induced 57 ± 8% DSI, and Tat did not alter DSI evoked by a 2-s depolarization (58 ± 7%; Fig. 2*C*,*D*; Table 1c). IPSC amplitude was not affected by 24-h Tat treatment (control = −541 ± 42 pA, Tat = −638 ± 91 pA; *t*_(25)_ = 0.97, *p *=* *0.34; Table 1b). Since not all inhibitory synapses contain CB_1_Rs, not all cells exhibit DSI; we found 29% of control cells responded to depolarization by exhibiting DSI (18 out of 62 cells). All synaptic pairs that failed to exhibit DSI were also insensitive to 1 μm Win-2, a potent and efficacious CB_1_R agonist, confirming a lack of presynaptic CB_1_Rs. Tat did not alter the percentage of cells that exhibited DSI, as Tat-treated cultures yielded responses in 32% of the cells (10 out of 31 cells). Overall, Tat does not alter DSI, in contrast to its marked inhibition of DSE, indicating a specificity for eCB signaling at excitatory but not inhibitory synapses.

### Tat inhibits MSE

**Figure 3.**
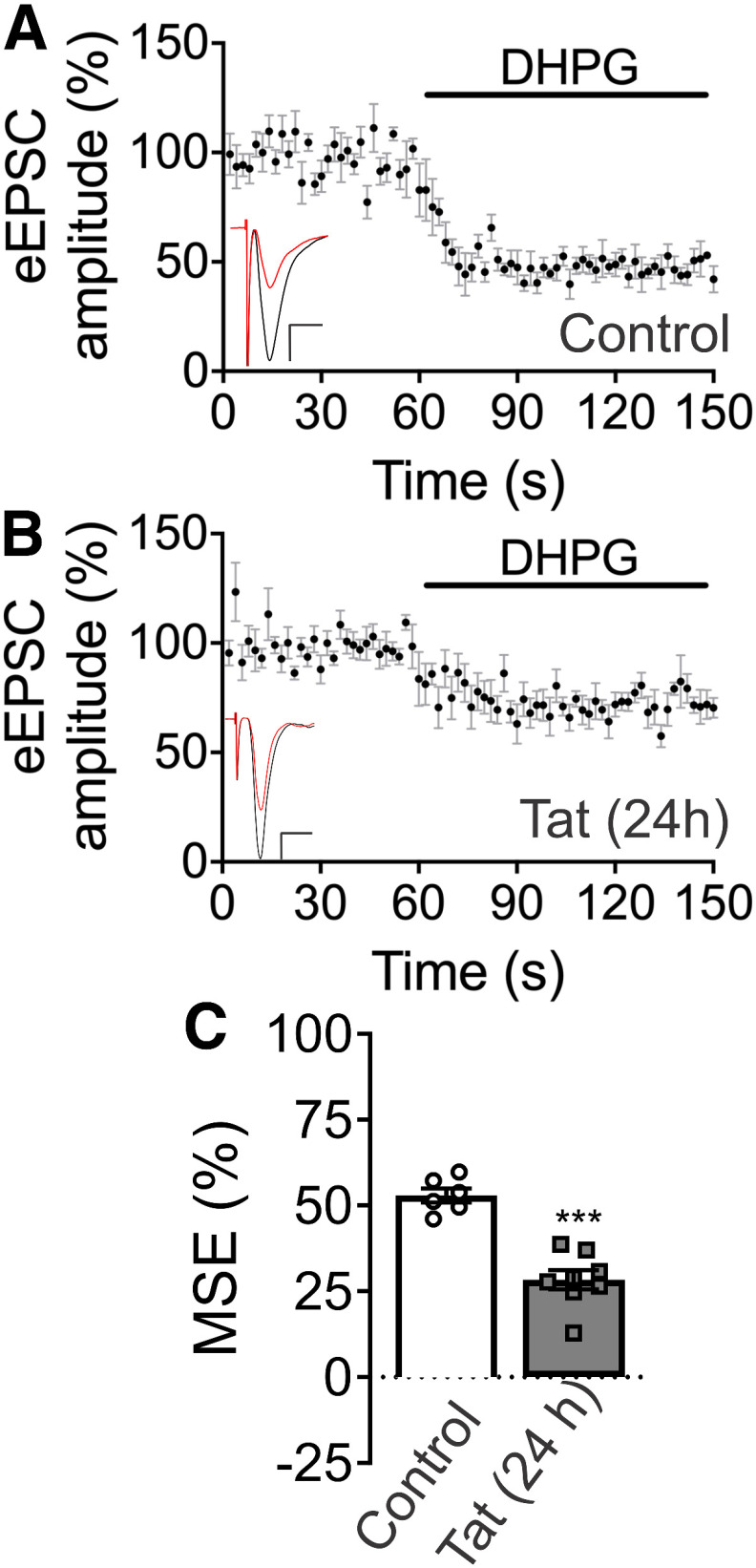
Exposure to HIV Tat inhibits MSE. ***A***, ***B***, EPSCs were evoked by 0.5-Hz stimulation of a presynaptic neuron with an extracellular electrode and recorded from a postsynaptic cell voltage-clamped at −70 mV. MSE was evoked by bath application of the selective group 1 mGluR agonist DHPG (1 μm). Plots show mean EPSC amplitudes as a percentage of baseline over time for (***A***) control and (***B***) 24-h Tat treatment groups. Insets show representative EPSC trace. Black: baseline, average of 30 responses before DHPG superfusion. Red: average of last 30 responses of DHPG superfusion. Scale bars: 10 ms (horizontal bar) and 50 pA (vertical bar). ***C***, Summary bar graph shows mean magnitude of MSE for control (open bar) and Tat-treated group (gray bar). Tat (50 ng/ml, 24 h) significantly reduced MSE (*t*_(12)_ = 6.577, ***p <* *0.0001; *t* test, *n *=* *6–8 cells/group). Data are represented as mean ± SEM.

We next determined whether sensitivity to Tat was dependent on the stimulus used to evoke 2-AG synthesis. MSE is evoked by activating postsynaptic G_q_-coupled receptors (Straiker and Mackie, 2007). Activation of group 1 mGluRs stimulates phospholipase Cβ (PLCβ) to produce diacyglycerol (DAG), the rate-limiting precursor for 2-AG synthesis (Murataeva et al., 2014). Bath application of the selective group 1 mGluR agonist DHPG (1 μm) inhibited EPSCs by 53 ± 2% (Fig. 3*A*). Tat treatment (50 ng/ml, 24 h) significantly reduced MSE to 28 ± 3% (Fig. 3*B*,*C*). We conducted a subsequent analysis to determine effect size and precision. Tat treatment for 24 h was estimated to decrease MSE relative to control by 25%. Using bootstrap resampling, we calculated the 95% CI for this effect as 19% to 31% (Table 1d). Thus, Tat impairs two forms of short-term synaptic plasticity mediated by the eCB system, both DSE and MSE.

### Tat does not alter diacylglycerol (DAG) synthesis

**Figure 4.**
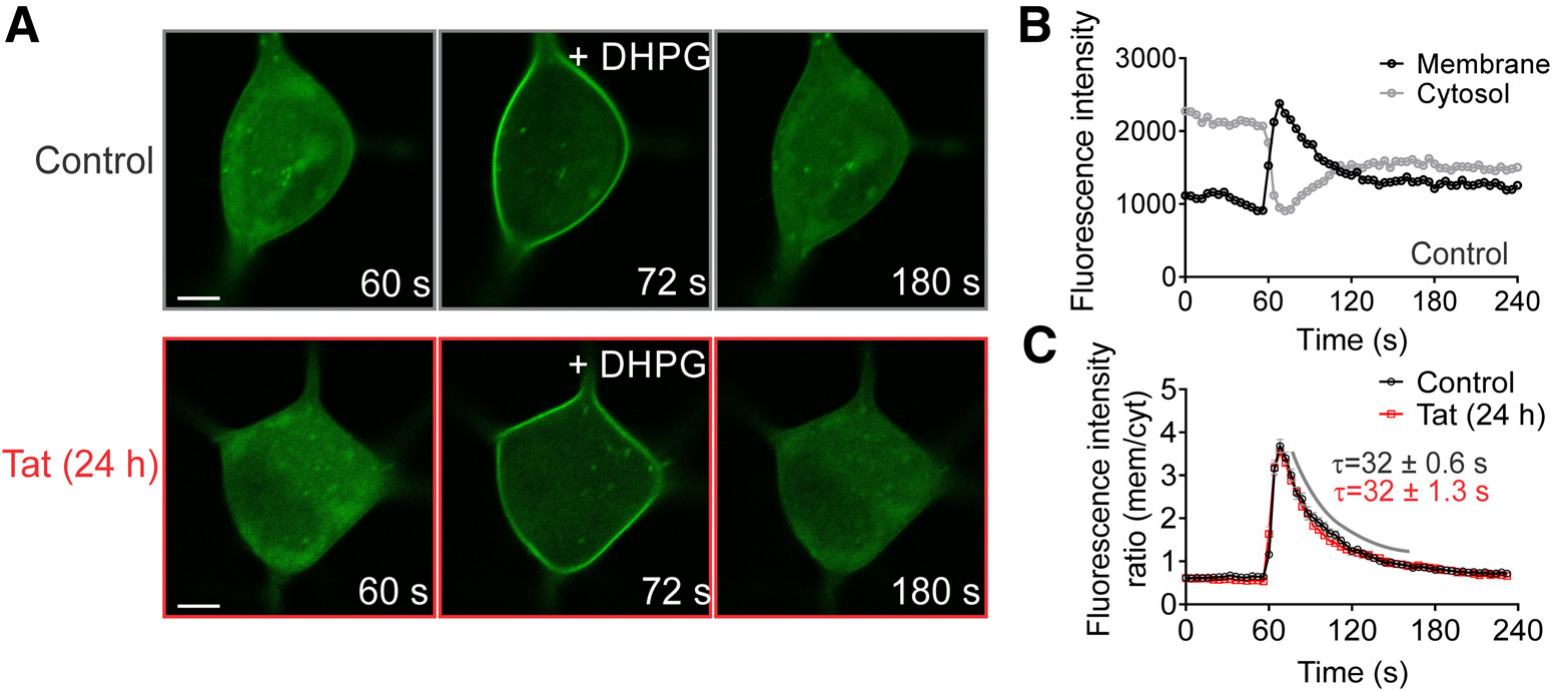
HIV Tat does not alter DAG synthesis. Cells expressing a fluorescent DAG bioprobe (pCMV-pKDC1ab-GFP) were stimulated by bath application of DHPG (1 μm). ***A***, Representative images of untreated cells (control, gray) and cells treated with 50 ng/ml Tat for 24 h (red). Scale bars = 5 μm**. *B***, Plot shows intensity values for regions of interest in the cytosol (gray) and membrane (black) monitored over time. ***C***, Ratio of membrane-to-cytosol fluorescence intensity plotted over time. Exponential functions were fitted to plots of peak DHPG response using a nonlinear, least-squares curve-fitting algorithm. The curve was fit by a logistic equation of the form fluorescence intensity ratio = yₒ + Ae^-(x-xₒ)/τ^. Tat did not significantly alter the translocation kinetics of the DAG biosensor, as indicated by no significant change in the peak response (control = 3.9 ± 0.23, Tat = 3.8 ± 0.15; *t*_(10)_ = 0.15, *p *=* *0.88, *t* test; *n *=* *6 cells/group) or decay time constant (τ; *t*_(10)_ = 0.31, *p *=* *0.76; *t* test). Data are represented as mean ratio ± SEM.

In a previous study, Tat was shown to inhibit PLC (Vitale et al., 2013), the enzyme that produces DAG; DAG is hydrolyzed to produce 2-AG, the eCB that mediates DSE and MSE. To determine whether Tat impairs production of DAG, cells were transfected with an expression vector (pCMV-PKDC1ab-GFP) for a fluorescent DAG bioprobe. Since DAG is a precursor for 2-AG, we used DAG as a proxy to assess changes in 2-AG synthesis. DAG production was stimulated by application of the selective group 1 mGluR agonist DHPG (1 μm). Upon stimulation, the DAG biosensor translocates from the cytosol to the membrane, where it binds DAG (Fig. 4*A*,*B*). Tat (50 ng/ml, 24 h) did not significantly alter the translocation kinetics of the DAG biosensor, as indicated by no significant change in the peak membrane-to-cytosol fluorescence intensity ratio (control = 3.9 ± 0.23, Tat = 3.8 ± 0.15) or decay time constant (τ; control = 32 ± 0.6 s, Tat = 32 ± 1.3 s; Fig. 4*A*,*C*; Table 1e). While this approach does not directly assess 2-AG production, these data do indicate that the production of DAG, the precursor for 2-AG synthesis, is not altered by Tat.

### Tat impairs CB_1_R function

**Figure 5.**
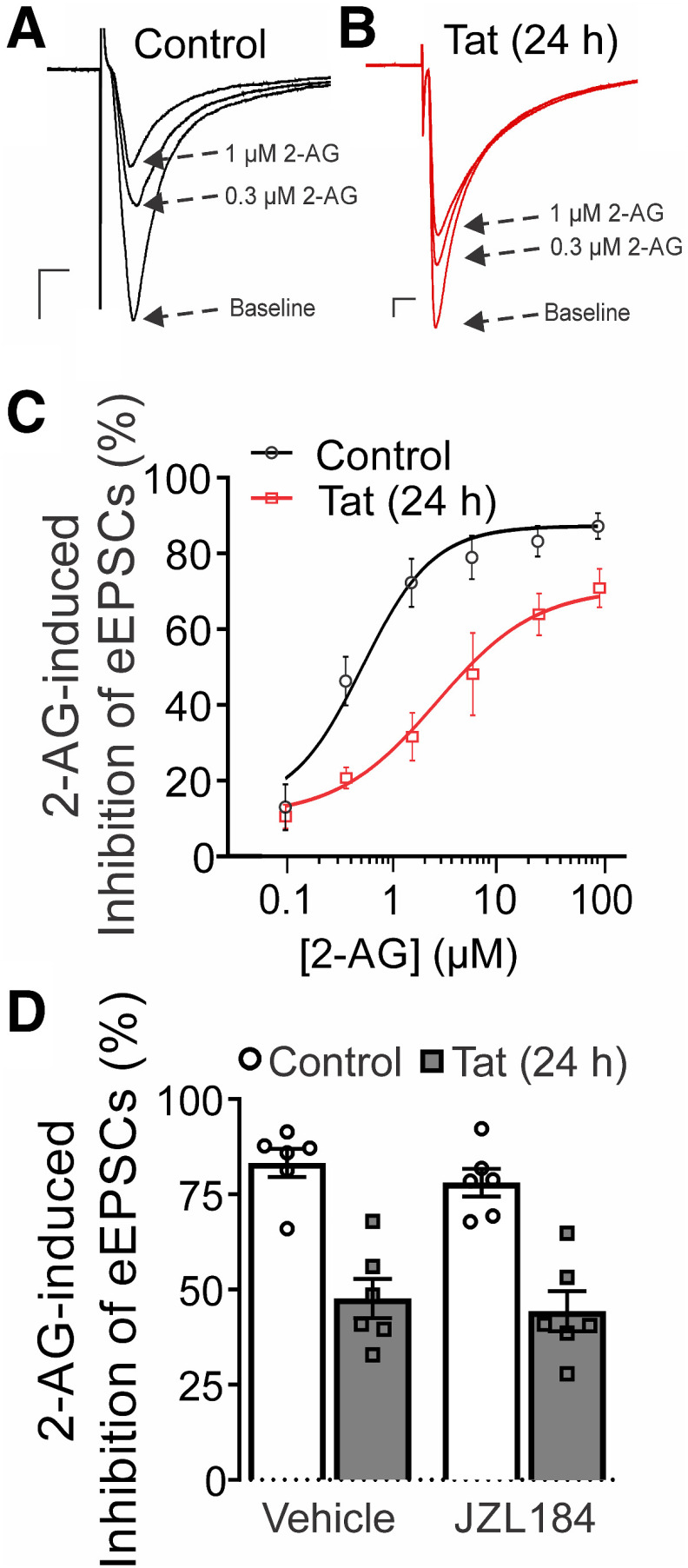
Exposure to HIV Tat reduces the potency of 2-AG-induced inhibition of EPSCs. ***A***, ***B***, Representative traces show EPSCs during baseline and in the presence of varying [2-AG] for (***A***) control and (***B***) Tat-treated groups (50 ng/ml, 24 h). Scale bars: 10 ms (horizontal bar) and 50 pA (vertical bar). ***C***, The concentration-response curve for 2-AG-mediated inhibition of EPSCs shows a rightward shift following Tat treatment. The curves were fit with a logistic equation of the form percentage inhibition = A_1_ + [(A_2_ – A_1_)/(1 + 10^(logxₒ-x)p^)], where xₒ = EC_50_, × = log[2-AG], A_1_ = % inhibition in the absence of drug, A_2_ = % inhibition at a maximally effective drug concentration, and p = slope factor. The following values were calculated using a nonlinear, least-squares curve fitting program: A_1_ = 13.1 for control, 10.4 for Tat; A_2_ = 87.1 for control, 70.3 for Tat; EC_50_ = 0.39 ± 0.07 μm for control, 1.6 ± 0.2 μm for Tat; *p* = 1.6 ± 0.04 for control, 1.1 ± 0.1 for Tat. The EC_50_ was significantly increased relative to control by treatment with Tat (*t* test; *t*_(37)_ = 4.91, *p *<* *0.0001), indicating reduced 2-AG potency. ***D***, Bar graph shows the mean magnitude of 0.2 μm 2-AG-mediated inhibition of EPSCs in control (open bars) and Tat-treated groups (gray bars) in cultures treated with vehicle or 1 μm JZL184 for 24 h. A two-way ANOVA found no interaction (JZL184 treatment × Tat treatment; *F*_(1,20)_ = 0.043, *p *=* *0.838, *n *=* *6 cells/group). Tat treatment had a significant effect on 2-AG-induced inhibition of EPSC amplitude (*F*_(1,20)_ = 59.01, *p *<* *0.0001). Data are represented as mean ± SEM.

Because the effects of Tat on eCB signaling were independent of postsynaptic stimulus and Tat did not appear to affect 2-AG synthesis, we next examined presynaptic components of the eCB system. We assessed changes in presynaptic CB_1_R function by measuring EPSC amplitude in the presence of various CB_1_R agonists. EPSCs were evoked at 0.5 Hz by stimulating the presynaptic neuron with an extracellular electrode. Exogenous 2-AG was bath applied at varying concentrations and found to inhibit EPSCs in a concentration-dependent manner (Fig. 5*A*,*C*). Following 24 h treatment with 50 ng/ml Tat, the concentration-response relationship for 2-AG-mediated inhibition of EPSCs was right-shifted (Fig. 5*B*,*C*), and the EC_50_ was significantly increased from 0.39 ± 0.07 μm (control) to 1.6 ± 0.24 μm, indicating reduced potency of 2-AG (Table 1f). The maximal inhibition produced by 2-AG trended lower in Tat-treated cells, as indicated by a shift in the asymptote of the sigmoidal curve fit from 87% in control to 70% after Tat treatment. We examined whether this Tat-induced shift in the 2-AG concentration-response relationship was the result of an increase in 2-AG metabolism by monoacylglycerol lipase (MGL) by treating the culture with the selective, irreversible MGL inhibitor JZL184 (1 μm; Long et al., 2009) for 24 h. We first confirmed that JZL184 effectively inhibited MGL. JZL184 treatment prolonged the duration of DSE, as indicated by a markedly slowed recovery from depolarization-induced inhibition (*n *=* *2). However, pretreating control or Tat-treated (50 ng/ml, 24 h) cultures with JZL184 did not affect 2-AG-mediated inhibition of EPSCs (Fig. 5*D*; Table 1f). These data suggest that breakdown of bath-applied 2-AG by MGL was not enhanced by Tat.

**Figure 6.**
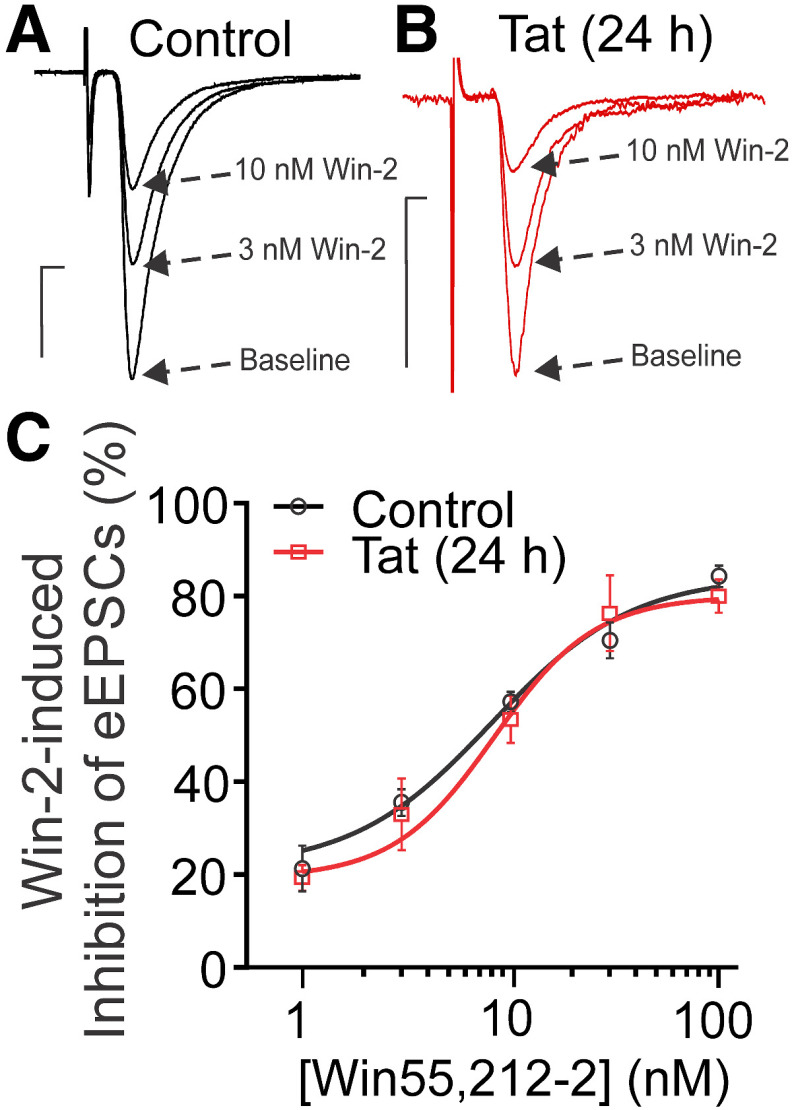
HIV Tat does not affect Win-2-mediated inhibition of EPSCs. Representative traces show EPSCs during baseline and in the presence of varying [Win-2] for (***A***) control and (***B***) Tat-treated groups (50 ng/ml, 24 h). Scale bars: 10 ms (horizontal bar) and 50 pA (vertical bar). ***C***, The concentration-response curve for Win-2-mediated inhibition of EPSCs was not affected by Tat treatment. The curves were fit with a logistic equation of the form percentage inhibition = A_1_ + [(A_2_ – A_1_)/(1 + 10^(logxₒ-x)p^)], where xₒ = EC_50_, × = log[Win-2], A_1_ = % inhibition in the absence of drug, A_2_ = % inhibition at a maximally effective drug concentration, and p = slope factor. The following values were calculated using a nonlinear, least-squares curve fitting program: A_1_ = 21.7 for control, 19.9 for Tat; A_2_ = 81.1 for control, 77.0 for Tat; EC_50_ = 7.8 ± 1.6 nm for control, 8.0 ± 1.1 nm for Tat; *p* = 2.5 ± 1.7 for control, 2.5 ± 1.2 for Tat. Tat treatment did not significantly affect the EC_50_ for Win-2-mediated inhibition of EPSCs (*t* test; *t*_(39)_ = 0.08, *p *=* *0.93).

**Figure 7.**
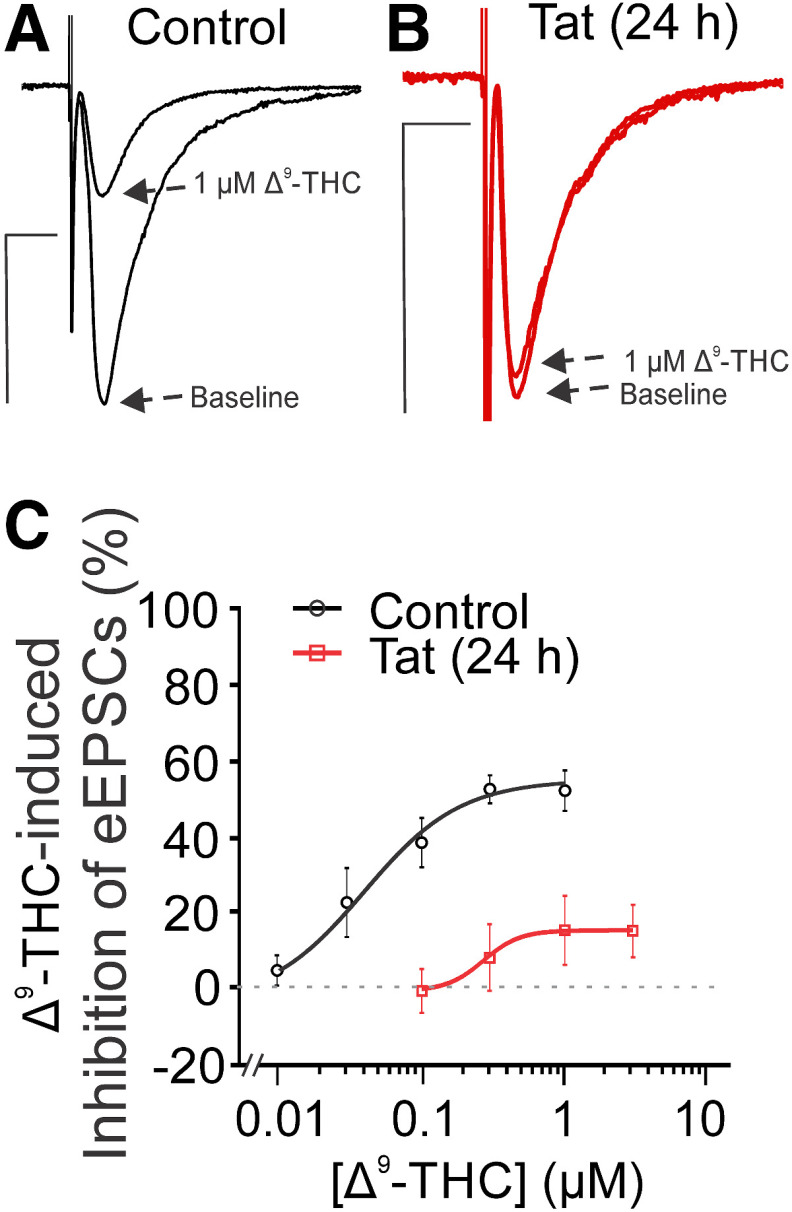
Exposure to HIV Tat reduces Δ^9^-THC-mediated inhibition of EPSCs. ***A***, ***B***, Representative traces show EPSCs during baseline and in the presence of varying [Δ^9^-THC] for (***A***) control and (***B***) Tat-treated groups (50 ng/ml, 24 h). Scale bars: 10 ms (horizontal bar) and 50 pA (vertical bar). ***C***, The concentration-response curve for Δ^9^-THC-mediated inhibition of EPSCs shows a decrease in potency and efficacy following exposure to Tat. The curves were fit with a logistic equation of the form percentage inhibition = A_1_ + [(A_2_ – A_1_)/(1 + 10^(logxₒ-x)p^)], where xₒ = EC_50_, × = log[Δ^9^-THC], A_1_ = % inhibition in the absence of drug, A_2_ = % inhibition at a maximally effective drug concentration, and p = slope factor. The following values were calculated using a nonlinear, least-squares curve fitting program: A_1_ = −4.4 for control, −0.8 for Tat; A_2_ = 54.6 for control, 15.3 for Tat; EC_50_ = 0.04 ± 0.03 μm for control, 0.27 μm for Tat; *p* = 1.3 ± 0.8 for control, 3.4 for Tat. The marked reduction in Δ^9^-THC-mediated inhibition following Tat exposure limited the quality of the curve fit precluding determination of error associated with the EC_50_. To determine the significance of the Tat effect, we performed a two-way ANOVA over the 0.1–1 μm THC concentrations that were tested in both control and Tat-treated cultures and found no interaction ([Δ^9^-THC] × Tat treatment; *F*_(2,19)_ = 0.088, *p *=* *0.92, *n *=* *4–5 cells/group). Tat treatment had a significant effect on Δ^9^-THC-induced inhibition of EPSC amplitude (*F*_(1,19)_ = 53.71, *p *<* *0.0001). Data are represented as mean ± SEM.

Interestingly, Tat did not alter the concentration-response relationship for Win-2-mediated inhibition of EPSCs (Fig. 6). The EC_50_ for Win-2 inhibition of EPSC amplitude was 7.8 ± 1.6 nm for control and 8.0 ± 1.1 nm after 24 h exposure to Tat (Table 1g). Win-2 is a highly efficacious agonist for cannabinoid receptors (Shen et al., 1996; Luk et al., 2004) and has been shown to elicit near maximal effect while occupying only 7.5% of CB_1_Rs (Gifford et al., 1999). Thus, the presence of spare receptors may explain the discrepancy. If spare receptors account for the insensitivity of Win-2-mediated inhibition of EPSCs to Tat treatment relative to 2-AG-mediated presynaptic inhibition, then we would predict that the inhibition produced by Δ^9^-tetrahydrocannabinol (Δ^9^-THC), the psychoactive ingredient in marijuana and a drug that acts as a partial agonist for CB_1_Rs (Shen and Thayer, 1999; Roloff and Thayer, 2009), to be especially sensitive to Tat exposure. Presynaptic inhibition mediated by G_i_-coupled GPCRs is attenuated at high stimulus frequencies (Bean, 1989), which is more pronounced for low-efficacy agonists such as Δ^9^-THC (Roloff and Thayer, 2009); thus, to study the effect of Δ^9^-THC we reduced the stimulus frequency to 0.1 Hz. Following Tat treatment, presynaptic inhibition produced by Δ^9^-THC was markedly reduced (Fig. 7; Table 1h). The inhibition of EPSC amplitude at a maximally effective concentration of Δ^9^-THC was reduced from 55% in control to 15% after Tat treatment. Overall, Tat impairs CB_1_R function and this loss-of-function is most evident by the attenuation of the responses to low-efficacy agonists such as Δ^9^-THC.

Several reports have shown that Tat produces synaptodendritic injury and cell death via a pathway initiated by Tat binding to low-density lipoprotein receptor-related protein (LRP; Eugenin et al., 2007; Kim et al., 2008; Shin and Thayer, 2013). Thus, we examined whether binding to LRP is necessary for Tat-mediated impairment of eCB signaling. We found that blocking LRP by pretreating with the selective antagonist receptor-associated protein (RAP) did not prevent Tat-mediated reduction of DSE. In untreated cells DSE was 51 ± 4% and was similar in the presence of 50 nm RAP (55 ± 6%). Tat treatment (50 ng/ml, 24 h) reduced DSE to 14 ± 12% and was not affected by RAP (12 ± 7%). A two-way ANOVA found no interaction (RAP treatment × Tat treatment; *F*_(1,16)_ = 0.156, *p *=* *0.698, *n *=* *4–6 cells/group) while Tat treatment had a significant effect on DSE (*F*_(1,16)_ = 30.94, *p *<* *0.0001; Table 1i), indicating that Tat acts via a mechanism independent of LRP.

### Tat does not affect adenosine A_1_ receptor (A_1_AR)-mediated presynaptic inhibition

**Figure 8.**
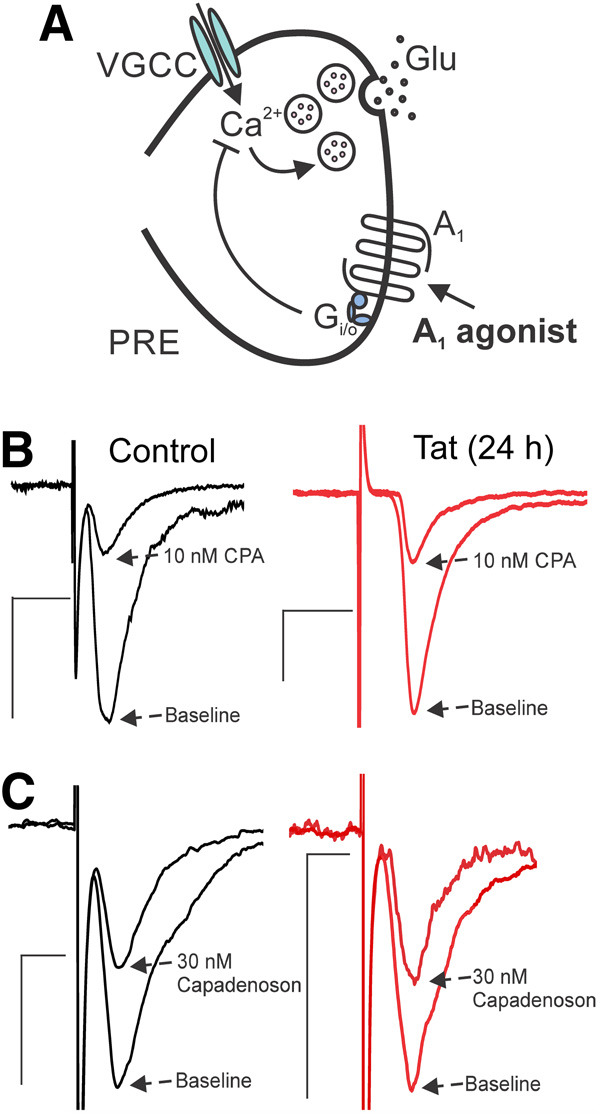
HIV Tat does not affect A_1_AR-mediated presynaptic inhibition. ***A***, Presynaptic terminal illustrating localization of A_1_ adenosine receptors. When these receptors are activated, glutamate transmission is reduced. ***B*,** Representative traces show EPSCs during baseline and in the presence of CPA (10 nm, selective A_1_AR agonist) for control and Tat-treated group (50 ng/ml, 24 h). Tat did not affect CPA-mediated inhibition of glutamate release (*t*_(10)_ = 0.416, *p *=* *0.686; *t* test). ***C***, Representative traces showing EPSCs during baseline and in the presence of the partial agonist capadenoson (30 nm) for control and Tat-treated group. Tat did not affect capadenoson-mediated inhibition of glutamate release (*t*_(10)_ = 0.187, *p *=* *0.856; *t* test). Scale bars: 10 ms (horizontal bar) and 50 pA (vertical bar).

To determine whether Tat acts selectively on CB_1_R, or whether it similarly affects other presynaptic G_i/o_-coupled receptors, we examined the effects of Tat on A_1_ARs. These receptors are localized on presynaptic terminals and modulate the release of neurotransmitters, including glutamate and GABA (Dunwiddie and Masino, 2001). We assessed whether Tat impairs A_1_AR function by monitoring EPSC amplitude in the presence of a selective A_1_AR agonist *N*^6^-cyclopentyladenosine (CPA; 10 nm; Hargus et al., 2009; Gonçalves et al., 2015) and partial agonist capadenoson (30 nm; Albrecht-Kupper et al., 2012). CPA inhibited EPSC amplitude by 74 ± 5% in control cultures and by 77 ± 5% in cultures treated with 50 ng/ml Tat for 24 h (Fig. 8*B*). The partial agonist capadenoson inhibited EPSC amplitude by 43 ± 8% in control cultures and by 41 ± 6% in Tat-treated cultures (Fig. 8*C*; Table 1j). Thus, Tat treatment does not affect inhibition of glutamate release initiated by selective A_1_AR agonists, indicating that A_1_AR function is not altered by Tat. These results suggest that Tat acts selectively to inhibit CB_1_R function and provides evidence that Tat exposure does not broadly suppress the function of all presynaptic G_i/o_-coupled receptors.

## Discussion

Treating rat hippocampal cultures with the HIV protein Tat inhibited DSE and MSE, two forms of short-term plasticity mediated by the eCB system. While Tat is a neurotoxin known to contribute to synaptodendritic damage in HAND (Li et al., 2009), this is the first report to show that Tat disrupts function of the eCB system. Our principal finding is that Tat impairs CB_1_R-mediated presynaptic inhibition at excitatory but not inhibitory terminals. This selective attenuation of eCB signaling may unbalance network excitability, with potentially significant effects on symptoms associated with HAND, the progression of neurological disease in people living with HIV, and the sensitivity of HIV-positive individuals to exogenous cannabinoids. Furthermore, the selective loss of eCB signaling at excitatory synapses has implications for the effects of neuroinflammatory stimuli on the eCB system.

Primary neuronal cultures provide an experimentally accessible system that exhibits synaptic changes that are a hallmark of HAND (Ellis et al., 2007; Eugenin et al., 2007; Kim et al., 2008, 2011a; Shin and Thayer, 2013; Raybuck et al., 2017; Hermes et al., 2018). Advantages of primary hippocampal cultures for this study include robust DSE and DSI, the ability to administer HIV Tat at specific concentrations with accurate timing, and the ease with which the culture can be treated with highly lipophilic cannabinoid receptor ligands. However, there are caveats to cell culture models. The rat primary cultures used in this study were derived from fetal neurons, and while they mature in culture, we cannot precisely match their stage of development to *in vivo* models. Furthermore, dissociated cultures lack the synaptic architecture found *in vivo*, precluding our ability to identify the specific synapses affected by HIV Tat in our model. Finally, the effects of chronic exposure to Tat produced in transgenic models cannot be replicated with short-lived primary cultures. Thus, the novel changes in the eCB system described here provide compelling evidence to extend these findings to chronic *in vivo* models to explore regional and developmental effects on synaptic networks.

Tat-induced attenuation of CB_1_R-mediated inhibition of glutamatergic, but not GABAergic neurotransmission, might result from several mechanisms. CB_1_Rs at inhibitory synapses are expressed at a much higher density relative to the density at excitatory terminals (Katona et al., 1999). Thus, Tat-induced loss of CB_1_R-mediated signaling at GABAergic terminals may not be sufficient to significantly impair eCB-mediated inhibition of GABA release. The importance of spare receptors in modulating eCB signaling is illustrated by comparing the failure of Tat to affect inhibition of glutamate release by the highly efficacious agonist Win-2, which elicits maximal effects while occupying only 7.5% of CB_1_Rs (Gifford et al., 1999), to the marked attenuation of the effects of the partial agonist Δ^9^-THC. Even when the duration of the depolarizing stimulus to evoke 2-AG production was reduced, Tat failed to affect DSI. Thus, following exposure to Tat, physiologically relevant eCB-mediated retrograde signaling remains functional at inhibitory terminals, but is impaired at excitatory synapses.

The rightward shift in the 2-AG concentration-response relationship produced by Tat treatment is reminiscent of CB_1_R desensitization (Kouznetsova et al., 2002; Sim-Selley, 2003; Lundberg et al., 2005; Daigle et al., 2008; Wu et al., 2008). Tat is a powerful excitotoxin; perhaps Tat-induced excitatory drive chronically activates the eCB system, desensitizing CB_1_Rs. The development of Tat-induced attenuation of CB_1_R function over the span of 4–48 h is consistent with gradual receptor downregulation. Alternatively, Tat can be taken up by glutamatergic neurons where it activates protein kinase C (Haughey et al., 2001), which is known to produce heterologous desensitization of CB_1_Rs (Garcia et al., 1998; Chu et al., 2010). The time course of the Tat effect also suggests that Tat does not simply bind to and occlude CB_1_Rs; direct receptor interaction would be expected to equilibrate more rapidly and also affect receptors on GABAergic terminals similarly to those on glutamatergic terminals. Tat can be internalized into neurons by binding to LRP, leading to synaptodendritic injury and cell death (Liu et al., 2000; Eugenin et al., 2007; Kim et al., 2008; Shin and Thayer, 2013). We found that Tat-mediated impairment of eCB signaling is independent of LRP. Tat elicits a range of toxic effects on neuronal networks, some of which are mediated by neuroinflammation (Lu et al., 2011; Chivero et al., 2017; Gonek et al., 2018; Thangaraj et al., 2018), leading us to speculate that Tat may impair eCB signaling through an indirect mechanism involving non-neuronal cells, possibly via the release of inflammatory cytokines (Kim et al., 2018). The degree to which this damage results from direct or adaptive responses to the presence of Tat is unclear.

In contrast to the loss of CB_1_R function described here, CB_1_R protein increased in infralimbic cortex from transgenic mice expressing Tat (Jacobs et al., 2019). The mPFC and hippocampus display differential sensitivity to Tat (Cirino et al., 2020), suggesting the effects of Tat on the eCB system may also exhibit regional differences. Xu et al. (2016) found that Tat expression occluded the effects of CB_1_R agonists. We found DSE to be an extremely sensitive assay for Tat-induced modulation of the eCB system. Thus, the failure of Tat to occlude DSI provides strong evidence that Tat does not affect CB_1_R-mediated presynaptic inhibition at GABAergic synapses in the hippocampal culture model. Tat failed to affect DSI evoked by three different stimulus strengths; this physiological method of graded CB_1_R activation essentially produces a 2-AG concentration-response. It is possible that Tat upregulates CB_1_Rs on GABAergic terminals while also decreasing CB_1_R levels on glutamatergic terminals. Tat elicits a neuroinflammatory response (Nath et al., 1999; Pu et al., 2003), and neuroinflammation selectively upregulates CB_1_Rs on GABAergic terminals (Chen et al., 2003; Feng et al., 2016), providing a precedent for this type of change in the eCB system. Alternatively, the loss of CB_1_R function at excitatory synapses described here may be an early event initiated by Tat, akin to the initial stages of exposure, and the gain-of-function at inhibitory terminals (Xu et al., 2016) may result from adaptations in the eCB system that occur during prolonged Tat expression. Thus, unique changes in the synaptic network may predominate at different disease stages.

Altered eCB signaling could affect many physiological processes relevant to HAND (Rodríguez de Fonseca et al., 2005). Using brain tissue from Alzheimer’s patients, autoradiography, *in situ* hybridization, and GTPγS binding studies have demonstrated reduced CB_1_R agonist binding, regionally discrete losses of CB_1_R mRNA expression, and less efficient coupling to G-proteins (Westlake et al., 1994; Ramírez et al., 2005). The selective loss of functional eCB signaling at excitatory terminals described here emphasizes the need to understand synapse-specific changes in the eCB system over the course of neurodegenerative disease. Antagonism of CB_1_R function elicits depression and anxiety-like behaviors (Moreira et al., 2009). If the loss of CB_1_R function on excitatory terminals we describe for hippocampal cultures also occurs in the prefrontal cortex (Rigucci et al., 2010), it might contribute to the production of these symptoms in HAND. The motor impairment produced by cannabinoids results from CB_1_R activation on both excitatory and inhibitory terminals in basal ganglia and cerebellum (Sañudo-Peña et al., 1999; Kishimoto and Kano, 2006), suggesting Tat-mediated motor effects could result from unbalanced eCB signaling (Kim et al., 2003). CB_1_Rs on glutamatergic terminals in the nucleus accumbens regulate the hedonic impact of food, while CB_1_Rs in the hypothalamus regulate the release of hormones that regulate appetite and energy expenditure (Lau et al., 2017). Thus, their loss could contribute to the loss of appetite and wasting observed in AIDS patients.

The eCB system protects against excitotoxicity (Shen et al., 1996; Shen and Thayer, 1998; Marsicano and Lutz, 1999; Nagayama et al., 1999; Chen et al., 2003; Li et al., 2012; Feng et al., 2016), including HIV-induced neurotoxicity (Kim et al., 2011a; Xu et al., 2017; Hermes et al., 2018; Zhang and Thayer, 2018). Thus, Tat-induced loss of neuroprotection may accelerate the development of neurological complications in HAND. There is precedent for changes in eCB signaling contributing to epileptogenesis following neuroinflammatory insult (Chen et al., 2003; Feng et al., 2016; Sugaya and Kano, 2018). Furthermore, in the Fmr1-knockout mouse model of fragile X syndrome, impaired eCB signaling contributes to synaptic plasticity defects that underlie impairments in episodic memory (Wang et al., 2018). Superfusion of amyloid-β_1-42_ onto brain slices from wild-type mice prolonged DSI, thus altering GABAergic transmission and possibly contributing to synaptic dysfunction in Alzheimer’s disease (Mulder et al., 2011).

While evidence linking impaired eCB signaling to disease progression is limited, there is considerable evidence demonstrating that enhanced CB_1_R signaling protects neurological function (Aymerich et al., 2018). In HAND models, cannabinoids protect dopaminergic neurons against damage from the HIV envelope glycoprotein gp120 (Hu et al., 2013), reduce Tat-induced release of nitric oxide (Esposito et al., 2002), and diminish Tat-induced increases in neuronal [Ca^2+^]_i_ (Xu et al., 2017). Boosting eCB tone by inhibiting eCB metabolism also attenuates HIV neurotoxicity; inhibition of MGL with the selective inhibitor JZL184 protects against excitatory synapse loss evoked by gp120 (Zhang and Thayer, 2018). Similarly, pharmacological inhibition of fatty acid amide hydrolase, the enzyme responsible for degrading the eCB anandamide, protects against Tat-mediated increases in [Ca^2+^]_i_ and dendritic damage (Hermes et al., 2018). Thus, cannabimimetics and drugs that inhibit eCB metabolism may slow the course of HIV neurotoxicity (Wu et al., 2019).

This study suggests that the converse may also be true; in the presence of HIV, impaired CB_1_R function at excitatory terminals may alter the response to exogenous cannabinoids. Because efforts to legalize medicinal and recreational marijuana are increasing access (Charilaou et al., 2017; Jones et al., 2018) and cannabis use is prevalent among people living with HIV (Hartzler et al., 2017), altered response to cannabinoids could impact many patients. We found a dramatic loss of Δ^9^-THC efficacy at excitatory synapses in the presence of Tat. However, CB_1_R signaling at inhibitory synapses was normal in the presence of Tat. Thus, the network effects of Δ^9^-THC could shift to an excitatory response due to CB_1_R-mediated suppression of GABA release without a corresponding decrease in glutamate release. This prediction is consistent with the increased miniature EPSC frequency observed in mice expressing Tat (Jacobs et al., 2019).

In conclusion, we have shown in an *in vitro* model of HIV neurotoxicity that the HIV protein Tat, an established contributor to HIV neurotoxicity, inhibits short-term eCB-mediated plasticity selectively at excitatory synapses. We speculate that this novel loss of CB_1_R function might contribute to excitotoxicity under neuroinflammatory conditions. Thus, our conclusions from this *in vitro* work provide a framework for future work assessing the status of the eCB system *in vivo* in neurodegenerative disease noting the duration of disease, the specific brain regions affected and, as shown here, the specific neurotransmitters involved.

## Synthesis

Reviewing Editor: Lindsay De Biase, University of California, Los Angeles Decisions are customarily a result of the Reviewing Editor and the peer reviewers coming together and discussing their recommendations until a consensus is reached. When revisions are invited, a fact-based synthesis statement explaining their decision and outlining what is needed to prepare a revision will be listed below. The following reviewer(s) agreed to reveal their identity: NONE. Note: If this manuscript was transferred from JNeurosci and a decision was made to accept the manuscript without peer review, a brief statement to this effect will instead be what is listed below.

The previous reviews of this manuscript acknowledge that this is a well-executed in vitro study with important observations regarding the impact of TAT on the endocannabinoid system. The experiments added at suggestion of reviewers have strengthened the manuscript. However, the abstract and discussion need to be further modified to acknowledge the limitations of the in vitro approach and to discuss additional experiments that could clarify if TAT-induced changes in endocannabinoid DSE and MSE contribute to neurological symptoms in vivo.

Please address the following concerns:

1) In the abstract: the final sentence should be modified to explicitly state that these in vitro findings suggest that similar effects of TAT could contribute to HAND in vivo.

2) The discussion needs to devote an entire paragraph to acknowledging possible differences between in vitro and in vivo neuronal and eCB function and point to critical future experiments that could reveal whether similar phenomena are observed in vivo. This would include acknowledging that cultured neurons generally exhibit more immature cellular phenotypes and may not fully reflect how mature neurons in vivo will respond to TAT insults. This should also include a discussion of any reported differences in the expression levels of CB1Rs in neurons in vitro and in vivo as well as any reported differences in the magnitude of endocannabinoid mediated DSE in vitro and in vivo.

3) It is not clear from the methods whether the authors used a range of stimulation intensities for each recorded neuron before settling on a stimulus intensity that evoked 50% max or 80% max EPSC or IPSC currents. If this was carried out, this should be described in the methods. In addition, the authors should use this data to comment on whether there were any baseline differences in EPSC or IPSC amplitude or the relationship between stimulus intensity and amplitude following TAT treatment but before inducing DSE or DSI.

4) Include in the introduction or results a description of the brain regions most affected by HAND as part of the rationale for using hippocampal cultures to investigate TAT-mediated effects on the eCB system.

5) Page 14 line 8 and Page 16 lines 11-14 discuss the implications of the LRP experiments and suggest that TAT acts through an “indirect” mechanism, potentially via glial cells. In the absence of experimental evidence showing that glial cells are involved, it seems more accurate to simply say that the effect of TAT on CB1R function is occurring through an “LRP-independent mechanism.”
